# Improvement of Contact Models by Finite Element Analysis for the Evaluation of Yeast Mechanical Properties

**DOI:** 10.3390/ma19132837

**Published:** 2026-07-03

**Authors:** Laisvidas Striska, Nikolajus Kozulinas, Rokas Astrauskas, Dainius Udris, Audrius Grainys, Sonata Tolvaisiene, Juste Rozene, Tomas Mockaitis, Arunas Ramanavicius, Inga Morkvenaite

**Affiliations:** 1 Department of Nanotechnology, Center for Physical Sciences and Technology, Sauletekio al. 3, 10257 Vilnius, Lithuaniaarunas.ramanavicius@chf.vu.lt (A.R.); 2 Department of Electrical Engineering, Vilnius Gediminas Technical University, Plytines g. 25, 10105 Vilnius, Lithuaniasonata.tolvaisiene@vilniustech.lt (S.T.); 3 Faculty of Mathematics and Informatics, Vilnius University, 01513 Vilnius, Lithuania; nikolajus.kozulinas@mif.vu.lt (N.K.); rokas.astrauskas@mif.vu.lt (R.A.); 4 Department of Mechatronics, Robotics, and Digital Manufacturing, Vilnius Gediminas Technical University, Plytines g. 25, 10105 Vilnius, Lithuania; 5 Department of Chemistry and Bioengineering, Vilnius Gediminas Technical University, Sauletekio al. 11, 10223 Vilnius, Lithuania; 6 Department of Physical Chemistry, Vilnius University, Naugarduko g. 24, 03225 Vilnius, Lithuania

**Keywords:** yeast, living cells, Young’s modulus, Hertz model, Sneddon flat-punch model, contact radius, contact evolution, finite element analysis, atomic force microscope

## Abstract

In this work, we extended our previous studies on the limitations of classical contact models from polymers to a biological system. We used yeast as a model system to investigate how contact evolution during indentation affects the accuracy of AFM-based determination of Young’s modulus. We proposed a practical correction framework for the classical Hertz and Sneddon flat-punch models to improve the extraction of mechanical properties from experimental data. Force-indentation curves were measured using a spherical (SPHERE) probe with a 2 μm radius and a flat (FLAT) probe with a 4 μm radius of plateau. The experimental results were analyzed using both corrected and uncorrected contact models, while a finite element analysis (FEA) model was used to determine the contact radius-indentation dependence. It showed that Young’s modulus estimated from AFM indentation using classical formulations is probe-dependent because the contact radius is inadequately described. By incorporating the FEA-derived effective contact radius into Hertz and Sneddon contact models, the same Young’s modulus was obtained for yeast with both probes and compared to reference values with other techniques. These findings establish contact evolution as a governing factor in AFM-based cell mechanics and provide a practical route toward robust, probe-independent, and more accurate determination of mechanical properties for living cells.

## 1. Introduction

Quantitative determination of the mechanical properties of living cells remains a challenging task in biomechanics, biophysics, and biomedical engineering [1,2].

Several techniques are used to assess the mechanical properties of cells and soft biological materials, including AFM, optical tweezers, Brillouin microscopy, micropipette aspiration, and optical microelastography [3,4]. Optical tweezers allow highly sensitive force manipulation at the microscale and nanoscale, but their use for whole-cell or structurally complex samples can be limited by trapping geometry, force range, and sample configuration [4]. Brillouin microscopy offers non-contact, label-free, three-dimensional mechanical contrast, although its signal is related to high-frequency longitudinal viscoelastic properties and hydration-dependent compressibility rather than directly to quasi-static Young’s modulus [3]. In contrast, AFM directly records local force-indentation curves and is widely used for nanomechanical characterization, but its accuracy depends strongly on probe geometry, calibration, contact-point determination, and the contact model used for fitting [4].

Cell stiffness is often associated with physiological state, environmental adaptation, and pathological processes [5,6,7]. However, despite the use of various experimental techniques and increasingly advanced characterization approaches, reported Young’s modulus values derived from atomic force microscopy (AFM) data for similar cell types remain highly scattered in the literature, sometimes differing by orders of magnitude [8,9]. This variability suggests that the difficulty lies not only in the biological complexity of living cells but also in the methodology used to determine their mechanical properties. The quantitative reliability of AFM depends not only on the experimental measurement itself, but also on the contact mechanics model used to interpret the force-indentation data. To date, no broadly accepted standardized framework has been established for robust and comparable determination of Young’s modulus in living cells from AFM data.

In most AFM studies, Young’s modulus is extracted using classical contact models, most commonly the Hertz model for spherical and Sneddon type for sharp and flat probes [10,11,12]. These models remain attractive because of their simplicity and widespread use in AFM data analysis routines. However, their assumptions are strongly idealized. They describe the sample as homogeneous, isotropic, linearly elastic, and effectively semi-infinite, while the contact geometry is defined in simplified form [13,14,15]. Such assumptions are rarely fully satisfied in living-cell measurements, where the specimen is structurally heterogeneous, finite in thickness, and mechanically complex, often showing nonlinear and viscoelastic behavior [5,6]. Consequently, Young’s modulus values obtained from AFM indentation may depend strongly on probe geometry, indentation depth, loading conditions, substrate effects, and finite-thickness (bottom-effect) artifacts, which have recently been identified as a significant source of systematic error in cell-mechanics measurements [16,17].

A major limitation of classical AFM-based mechanical analysis is the insufficient representation of the real probe-sample contact [18,19]. During indentation, the contact evolves continuously, but the effective contact radius is not measured directly and is only indirectly or inadequately represented by conventional analytical models [20,21,22,23,24,25]. This issue is especially important for living cells, where the contact cannot be assumed to have linear behavior [26,27,28]. In addition, the actual probe geometry may differ from nominal manufacturer values because of fabrication tolerances or wear [29]. Under such conditions, inaccuracies in contact description can translate into substantial errors in the final Young’s modulus. Therefore, one of the key unresolved problems in AFM-based cell mechanics is not only the biological variability, but also the lack of a physically reliable description of the evolving indentation contact. This problem formed the basis of our previous studies on well-characterized polymer materials, in which we investigated the limitations of classical contact models in AFM analysis and demonstrated that the contact radius inferred by idealized formulations can be substantially overestimated, leading to systematic and probe-dependent errors in determining Young’s modulus [18,19]. To address this limitation, we introduced an effective contact radius, R_eff_, into Hertz-based mechanical contact models and determined its evolution using finite element analysis (FEA) [19]. That methodology was validated using probes with substantially different geometries, including sharp pyramidal and flat-ended indenters. It showed that accounting for contact evolution can significantly reduce probe-dependent inconsistencies in the extracted modulus. The present study is a direct continuation of that line of research and extends it from polymers to living cells.

This study aims to investigate how contact evolution during cell indentation affects the final Young’s modulus, identify the major analytical factors that lead to error, and evaluate practical improvements to the classical Hertz and Sneddon flat-punch models using FEA-resolved contact evolution. To make the limitations of these models explicit, two distinct probe geometries were selected: a spherical probe with a 2 μm radius (SPHERE) and a flat probe with a 4 μm radius of plateau (FLAT) (Table 1). These probe geometries were chosen to represent substantially different contact conditions and to challenge two commonly used analytical descriptions.

The study provides a practical route toward more robust, probe-independent, and physically better determination of Young’s modulus in living cells.

## 2. Materials and Methods

### 2.1. The Yeast Sample Preparation

Saccharomyces cerevisiae was selected as a model system because it is easy to cultivate, simple to prepare, and mechanically robust, enabling reproducible AFM measurements over extended experimental periods. *S. cerevisiae*, set Y10001 culture, was prepared by the methodology described in [32]. The yeast cultures were inoculated onto 5% YPD agar Petri dishes using a deep-frozen library sample and then incubated at +30 °C for 48 h. For further preparation of yeast cells, autoclaved 5% YPD broth vials were inoculated using the grown colonies, and cultivation was proceeded for an additional 24 h in a 30 °C incubator, shaking at 200 revolutions per minute. After the YPD broth vials were fully inoculated, the cell-containing media were transferred into 1.5 mL Eppendorf vials for the separation and washing of yeast cultures, then centrifuged at 5500 RPM for 5 min. The supernatant was discarded, and the samples were replaced with PBS. The samples were then homogenized and centrifuged for an additional 5 min. This procedure was performed a total of three times. After discarding the remaining supernatant, the pellet was weighed and diluted with PBS to obtain a 1 g/mL yeast solution.

### 2.2. Finite Element Modeling

**Figure 1:**
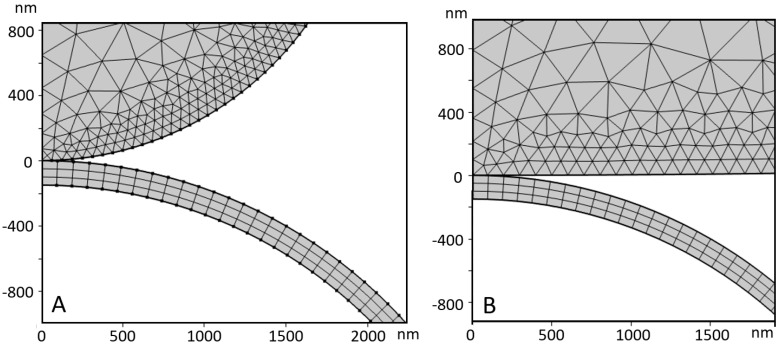
FEA model: mesh distribution. (**A**) SPHERE, (**B**) FLAT.

Finite element simulations were performed to analyze the indentation of spherical and flat nanoindenter shape architectures of yeast cells during AFM experiments (Figure 1). A stationary axisymmetric model in COMSOL Multiphysics 5.5 (Comsol AB, Stockholm, Sweden) was used. The indenter was modeled as a rigid silicon nitride material by prescribing displacement along the z-axis. The cell (R = 3 μm) consists of an external chitin layer and an internal cytoplasm. The internal volume was modeled using the Enclosed Cavity module with an incompressible fluid. The cell wall was described as a hyperelastic material using the Saint-Venant-Kirchhoff model with a Young’s modulus of 1.1 MPa and a Poisson’s ratio of 0.49. A Poisson’s ratio of 0.49 was used to approximate the yeast cell wall as a nearly incompressible soft biological material, consistent with common assumptions in AFM-based cell-mechanics analysis [33]. The chitin layer is 150 nm thick. The interaction between the indenter and the cell wall surface was modeled using the augmented Lagrangian contact method. Contact was assumed to be frictionless. The indenter was meshed with triangular elements, while the external layer was meshed with quadrilateral elements. Meshing the cell’s internal volume was unnecessary because the Enclosed Cavity module in COMSOL was used. The density of the indenter mesh was increased near the contact area by specifying a denser mesh distribution. These simulations predicted the contact radius, deformation field, and force-indentation behavior. This allowed for a direct comparison with Hertzian analytical predictions and atomic force microscopy (AFM) experimental data.

### 2.3. Atomic Force Microscopy Experiments

We used the BioScope II AFM and an optical microscope developed by Veeco Instruments Ltd. (Santa Barbara, CA, USA) to measure force-indentation curves with two probes of distinct geometries (Table 1). Spring constant calibration of the AFM cantilevers was performed using the thermal-noise (“Thermal Tune”) method on the Bruker BioScope II/NanoScope V system under ambient (air) conditions [34]. Each measurement was performed at least 25 times.

Young’s modulus values were determined from AFM force spectroscopy data using the classical Hertz model (Equation (1)) for the spherical indenter. The same force spectroscopy datasets were evaluated using our proposed corrected formulations (Equations (2) and (3)). The classical Sneddon flat-punch model (Equation (4)) and the modified one (Equation (5)) were used to evaluate data obtained with the flat indenter.

### 2.4. Calculations

The Young’s modulus *E_nom_* was calculated by fitting the Hertz model [35]:

(1) $$F=\frac{4}{3}\frac{E_{nom}}{\left(1-\upsilon^{2}\right)}\sqrt{R}\delta^{3/2}$$

where *F* is the indentation force, *E_nom_* is Young’s modulus, *ν* is Poisson’s ratio (0.49), *R* is the probe radius, and *δ* is the indentation.

In the modified Hertz model, we used *R_eff_* instead of *R*. *R_eff_* was calculated [8]:

(2) $$R_{eff}=\frac{a^{2}}{\delta }$$

where *a* = *P*1·*δ*/(*P*2 + *δ*) obtained from FEA data.

Contact radius *a* represents the contact radius obtained from the FEA model as a function of indentation depth δ. The expression $a=P_{1}\delta /(P_{2}+\delta )$ is not derived from classical contact mechanics and should therefore be regarded as a phenomenological fitting function. It was introduced to provide a simple analytical description of the contact radius evolution as calculated by FEA for the SPHERE probe. The parameters $P_{1}$ and $P_{2}$ are fitting coefficients and do not represent independent physical constants. The fitted function was used only within the indentation range investigated in this study and was subsequently inserted into the modified Hertz formulation to account for the FEA-predicted contact evolution.

Equation (1) with *R_eff_* becomes:

(3) $$F=\frac{4}{3}\frac{E_{eff}\cdot P1\cdot \delta^{2}}{\left(1-\upsilon^{2}\right)\cdot (P2+\delta )}$$

where *P*1 = 8.22343 × 10^−8^ m; *P*2 = 2.72913 × 10^−8^ m.

For a rigid flat cylindrical punch of radius *a* indenting an elastic half-space [36]:

(4) $$F=\frac{2{\cdot E}_{nom}}{\left(1-\upsilon^{2}\right)}\delta a$$

Modified flat-punch equation was obtained by inserting *a* = (*P*1 + *P*2·*δ*) obtained from FEA data. Equation (4) becomes:

(5) $$F=\frac{2\cdot E_{eff}}{\left(1-\upsilon^{2}\right)}(P1\cdot \delta +P2\cdot \delta^{2})$$

where *P*1 = 7.27553 × 10^−11^ m; *P*2 = 3.22854.

### 2.5. Young’s Modulus Evaluation Procedure

**Figure 2:**
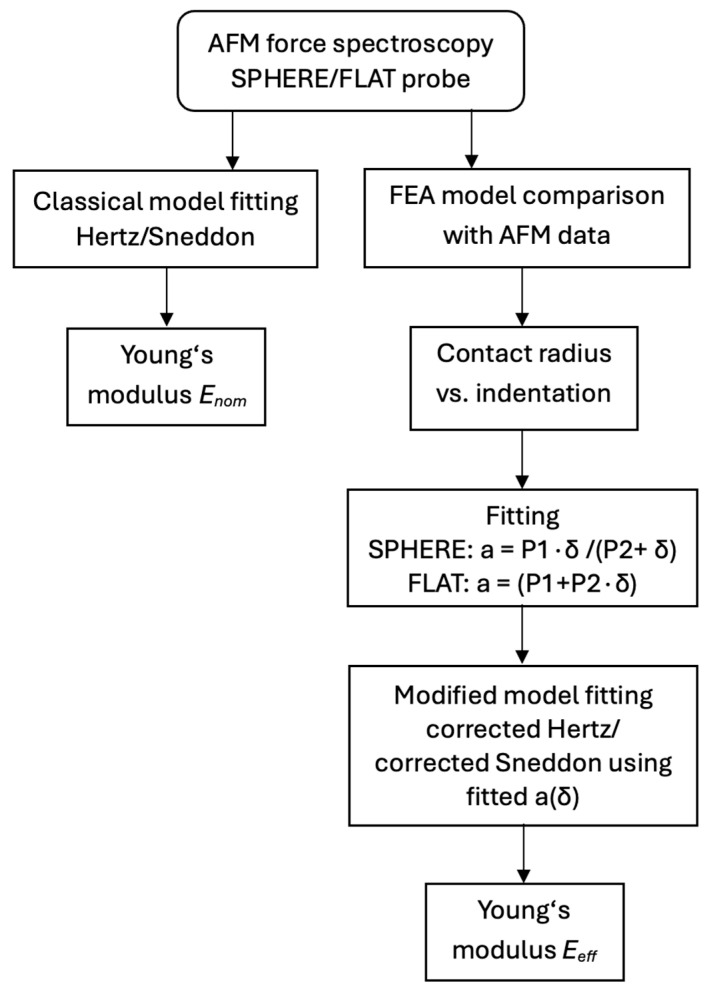
Young’s modulus determination algorithm.

AFM force spectroscopy was performed using SPHERE and FLAT probes (Figure 2). The raw force curves were converted to force-indentation curves using AFM software. Experimental force-indentation data from SPHERE fitted using the classical Hertz model (Equations (1)–(3)). The classical Sneddon flat-punch model was used for the FLAT probe (Equations (4) and (5)). FEA models for different geometries were compared with AFM force-indentation data to verify that the numerical simulation reproduced the measured indentation behavior. After validation, the FEA results were used to determine the dependence of the contact radius on indentation. This dependence was fitted by analytical expressions: a = P1δ/(P2 + δ) for SPHERE and a = (P1 + P2δ) for FLAT probe.

The fitted contact radius vs. indentation dependence, a(*δ*), was inserted into the corresponding modified Hertz or Sneddon formulation. The same experimental force-indentation curves were then fitted using corrected models to obtain effective Young’s modulus values.

## 3. Results and Discussion

Yeast cells were used as the test specimen to evaluate how contact evolution during AFM indentation affects the determination of mechanical properties. Finite element analysis (FEA) was performed for two distinct AFM probe geometries, namely a spherical indenter (SPHERE) and a flat-ended indenter (FLAT), in order to simulate the indentation experiment and to investigate the evolution of the probe-cell contact under the applied loading conditions. In both cases, the indenter was modeled as a rigid body that interacted with a deformable yeast cell. The simulations provided both the force-indentation response and the evolution of the contact radius during indentation.

The FEA model in this study plays a twofold role. First, it was used to reproduce the experimental AFM force spectroscopy response, thereby validating the numerical model under the experimental conditions. Second, once validated, it was used to analyze the real contact evolution for both probe geometries and to compare it with the contact-radius assumptions inherent in the classical Hertz and Sneddon flat-punch models. This comparison allows direct evaluation of how inaccuracies in contact-radius estimation influence the Young’s modulus obtained from AFM force spectroscopy data.

Based on this analysis, Young’s modulus values were extracted using the classical Hertz model for the spherical indenter and the classical Sneddon flat-punch model for the flat indenter. The same datasets were then re-evaluated using our proposed corrected formulations, in which the classical models were updated according to indenter type by incorporating a more realistic contact-radius evolution derived from FEA. This approach is particularly relevant for AFM users working with large-radius spherical or flat indenters.

### 3.1. Determination of Young’s Modulus by SPHERE

**Figure 3:**
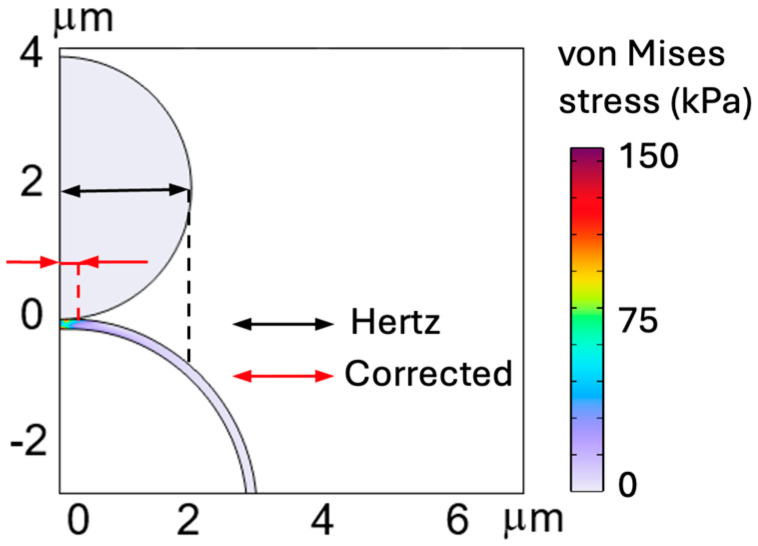
Schematic representation of the effective contact radius and the nominal sphere radius R used to calculate the Hertz contact radius.

Figure 3 schematically illustrates the difference between the Hertz model’s assumed contact radius and the real contact radius obtained from FEA. This visualization helps to clarify why FEA is a useful tool in the present study: it not only simulates the experiment but also reveals the actual contact evolution that classical analytical models fail to capture.

**Figure 4:**
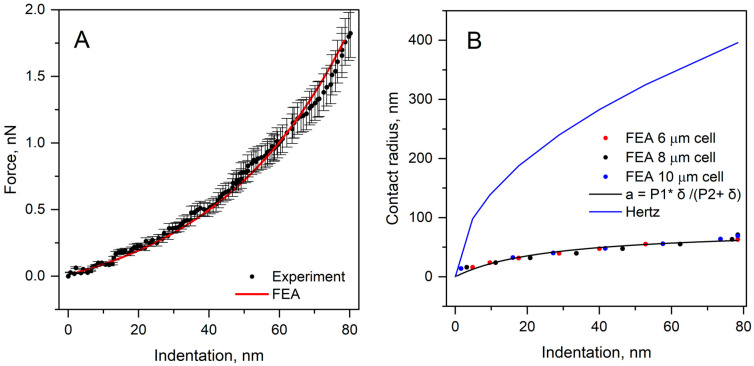
(**A**) Comparison of the experimental AFM force-indentation response and the FEA simulated response for the SPHERE probe. (**B**) Evolution of contact radius with indentation depth for the spherical indenter. FEA data obtained from three different cell sizes are presented. The black curve represents the fitted effective contact-radius relation. Equation a = P1·*δ*/(P2 + *δ*) parameters are: P1 = 8.22343·10^−8^ m; P2 = 2.72913·10^−8^ m.

A good agreement between the experimental AFM force-indentation curve and the corresponding FEA result for the SPHERE probe was obtained, indicating that the numerical model adequately reproduces the indentation behavior of the yeast cell (Figure 4A). The FEA results clearly show that the real contact evolution differs substantially from the contact radius predicted by the classical Hertz formulation (Figure 4B). At an indentation depth of 80 nm, the effective contact radius obtained from FEA is 62.8 nm. In contrast, the classical Hertz model predicts a contact radius of 396 nm at 80 nm indentation. Thus, even for the spherical indenter, the Hertzian formulation markedly overestimates the contact radius under the present experimental conditions. Because Young’s modulus estimated from AFM indentation using the Hertz model is highly sensitive to the assumed contact radius [18,19], this overestimation leads directly to underestimation of the modulus. To describe the FEA-derived contact evolution in analytical form, the effective contact radius was fitted using the expression a = P1·δ/(P2 + δ) with parameters P1 = 8.22343·10^−8^ m; P2 = 2.72913·10^−8^ m.

The model with a 6 µm cell diameter represents the experimentally observed yeast cell size used in the AFM measurements, while additional FEA simulations with 8 µm and 10 µm cell diameters were included to evaluate the influence of cell-size variation on the evolution of contact radius (Figure 4B). The results showed that the contact-radius values obtained for different cell diameters were very similar, indicating that, for the SPHERE probe and the analyzed indentation range, cell size had only a limited effect on the calculated contact radius.

**Figure 5:**
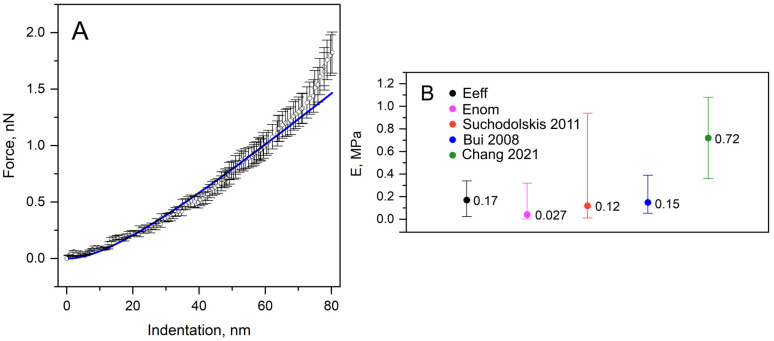
(**A**) Experimental force-indentation response for the SPHERE probe fitted with the modified Hertz-based formulation using the effective contact radius (R_eff_) by Equation (3). Yielding *E_eff_* = 0.17 MPa. (**B**) Young’s modulus obtained by SPHERE. *E_ref_* is the reference values from [37,38,39], *E_nom_* obtained by fitting Equation (1) with *R* = 2 μm.

A modified Hertz-based formulation provides a more realistic representation of the evolving mechanical contact during indentation than the classical Hertz assumption. The fitted curve shows good agreement with the experimental data (Figure 5A). It yields an effective Young’s modulus of 0.17 MPa, which is in good agreement with the reference values of E_ref_ (Figure 5B). Using the classical Hertz model with the nominal spherical geometry yielded a Young’s modulus of 0.027 MPa. The result demonstrates that the classical model significantly underestimates the modulus because it overestimates the contact radius. The corrected formulation (Equation (3)) therefore provides a more consistent estimate of the yeast cell’s mechanical properties.

Together, these results demonstrate that accounting for the evolution of effective contact is essential to obtaining robust mechanical property values from AFM indentation of living cells.

### 3.2. Determination of Young’s Modulus by FLAT

**Figure 6:**
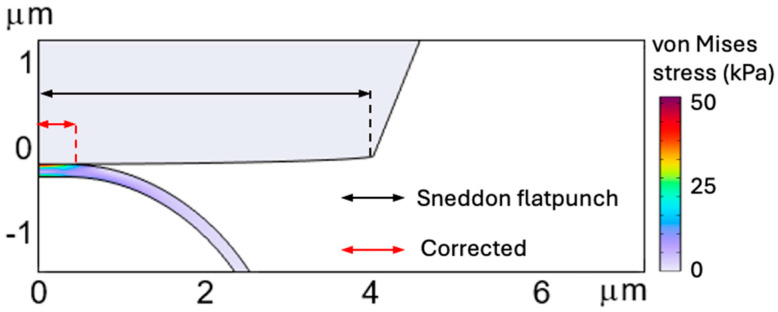
Visualization of contact radius: Sneddon flat-punch assumption vs. FEA.

The difference between the constant contact radius assumed by the classical Sneddon flat-punch model and the smaller, evolving contact radius obtained from FEA is visualized in Figure 6. This comparison highlights the source of the error in determining the modulus. Because the classical model assumes an excessively large contact area from the onset of indentation, it leads to a severe underestimation of Young’s modulus.

**Figure 7:**
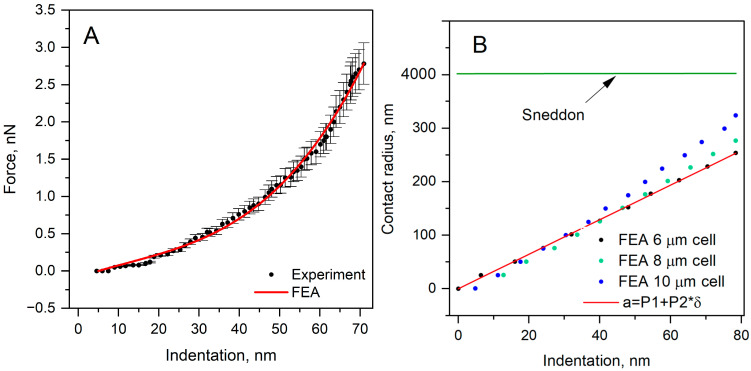
(**A**). Force–indentation curve fitted with FEA model. (**B**). Contact radius dependence on indentation using classical Sneddon’s flat-punch and FEA models, using three different cell diameters. Equation a = (P1 + P2·*δ*) parameters are: P1 = 7.27553·10^−11^ m; P2 = 3.22854.

For the FLAT probe, the FEA results showed good agreement with the experimental AFM force–indentation data, confirming that the numerical model adequately reproduces the indentation response of the yeast cell under the present experimental conditions (Figure 7A). This agreement supports the use of FEA not only for the simulation of the experiment, but also for the investigation of the real contact evolution during indentation.

**Figure 8:**
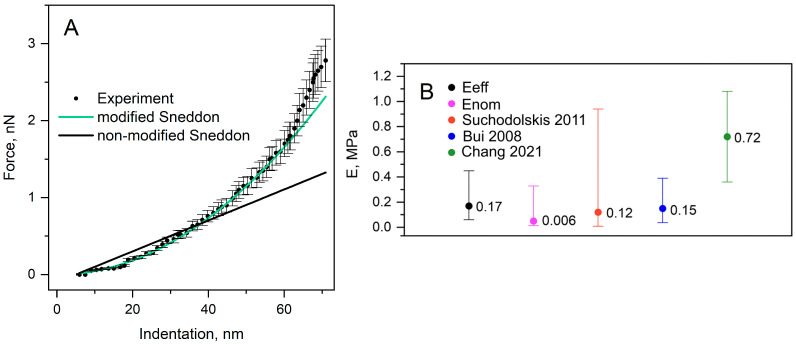
(**A**) Force dependence on indentation with FLAT probe. Modified Sneddon flat-punch model (Equation (5)) fitted to experimental data with *E_eff_* = 0.17 MPa, unmodified (classical) Sneddon flat-punch model fitted with *E_nom_* = 0.006 MPa. (**B**) Young’s modulus of FLAT. *E_ref_* is the reference values from [37,38,39], *E_nom_* is obtained by fitting Equation (4) to experimental data with *a* = 4 μm.

The experimental force-indentation response obtained with the FLAT probe is clearly nonlinear (Figure 7A). This behavior indicates that the indentation process cannot be adequately described by the classical Sneddon flat-punch formulation, which assumes an idealized, constant contact condition and thus yields a fundamentally different linear response. The real probe-sample contact does not instantaneously engage the full nominal flat-punch radius (Figure 7B). Instead, the effective contact radius evolves progressively with indentation depth and remains significantly smaller than the nominal flat-punch radius over the indentation range. Thus, the contact evolution is not constant, as assumed by the classical Sneddon’s flat-punch model, and this mismatch is directly reflected in the nonlinear experimental force-indentation response (Figure 7A). To represent the FEA-derived contact evolution in analytical form, the effective contact radius was fitted using the expression a = (P1 + P2∙δ), where $P_{1}$ has units of length and $P_{2}$ is dimensionless. In the present configuration, $P_{1}$ was close to zero, indicating that the effective contact radius increased approximately proportionally with indentation depth. This result shows that the nominal flat-punch radius was not fully engaged during the analyzed indentation range and that the classical assumption of a constant flat-punch contact radius is therefore not physically appropriate. The fitted relation should be regarded as a geometry-specific phenomenological approximation valid only for the investigated probe-cell configuration and indentation range. This relation provides an adequate approximation of the effective contact radius evolution within the studied indentation range and allows us to modify Sneddon’s flat-punch formulation, which becomes nonlinear (Figure 8A). This clearly demonstrates that the classical assumption of constant contact radius is not physically appropriate for AFM indentation with a nominally flat indenter.

Additional simulations with larger yeast cell diameters, 8 μm and 10 μm, were performed to evaluate whether natural variation in cell size could influence contact-radius evolution and, consequently, the Young’s modulus obtained from the modified model (Figure 7B). This is important because yeast cells are not identical in size, and differences in cell geometry may affect local deformation under the same indentation conditions. The results showed that larger cell radii yielded comparable contact-radius values at low indentation depths, whereas the contact radius increased with larger cell radii as indentation increased. These findings indicate that cell size can contribute to variation in contact-radius estimation, especially at higher indentation depths, but the overall dependence remains approximately linear for the FLAT probe. Therefore, the additional models confirm that the proposed linear approximation is suitable for the indentation range studied, while also indicating that cell geometry should be considered when applying the model to cells of different sizes.

The modified Sneddon flat-punch formulation fitted to the experimental force–indentation data (Figure 8A). In contrast to the classical approach, the corrected model more realistically captures the actual indentation behavior because it accounts for evolving mechanical contact rather than imposing a constant-radius condition from the start of loading. This result demonstrates that the main limitation of the classical flat-punch formulation is its unrealistic assumption of a constant contact radius.

Fitting the classical Sneddon’s flat-punch model (Figure 8A, non-modified Sneddon), the Young’s modulus of the yeast cell was estimated as *E_nom_* = 0.006 MPa (Figure 8B). In contrast, the modified formulation (Figure 8A, modified Sneddon) incorporating the FEA-derived effective contact radius yielded a Young’s modulus of *E_eff_* = 0.17 MPa (Figure 8B). The same Young’s modulus value was obtained for SPHERE with the modified Hertz formulation. Thus, the classical models yield a markedly underestimated Young’s modulus, demonstrating that contact radius overestimation is the main source of error.

Reported Young’s modulus values for Saccharomyces cerevisiae span a very broad range, depending on the experimental conditions and analysis method, from 0.12 to 0.32 MPa in AFM studies of chemically modified cells [37], and 0.15–0.24 MPa in aqueous environments [38], to substantially higher values such as 0.72 ± 0.36 MPa (with flat tip, radius of 2.5 μm) and 5.09 ± 1.51 MPa reported using other AFM-based approaches [39]. This pronounced inter-laboratory variation illustrates one of the main unresolved issues in AFM-based determination of cell mechanical properties. The value obtained in the present study (~0.17 MPa) falls within the lower bound of the reported range and suggests that part of the observed variability between studies may originate from the use of classical contact models that do not adequately capture the evolution of the contact radius during indentation.

Recent studies have highlighted another important limitation of AFM-based cell mechanics, namely, finite-thickness and bottom-effect artifacts, which arise when the deformation field extends to the underlying substrate [16,17]. Under such conditions, the measured response may reflect not only the cell itself but also the supporting surface, leading to overestimation of Young’s modulus. This effect becomes increasingly important as indentation depth increases and sample thickness decreases. While these studies emphasize substrate-induced stiffening as a source of bias, the present work identifies a different mechanism related to the description of probe–sample contact geometry. Our FEA results demonstrate that classical Hertz and Sneddon formulations substantially overestimate the contact radius, resulting in an underestimation of Young’s modulus. Therefore, the two effects act in opposite directions: bottom-effect artifacts tend to increase the apparent modulus, whereas contact-radius misestimation decreases it. Together, these findings indicate that accurate AFM-based mechanical characterization requires both appropriate treatment of finite-thickness effects and a realistic description of contact evolution. Future developments of AFM contact mechanics models should consider the combined influence of these factors to improve the reliability and comparability of reported Young’s modulus values. As shown here, misestimation of the contact radius can strongly affect the extracted Young’s modulus. It is therefore important to note that, in some published reports, the error bars are comparable to, or nearly as large as, the modulus values themselves, which may further indicate the strong sensitivity of the result to contact-radius estimation.

From a practical perspective, this corrected approach provides a solution for AFM users who aim to determine cell mechanical properties using flat indenters. This workflow can therefore be proposed: first, determine the contact radius-indentation relation using FEA for the selected cantilever geometry; second, fit this relation with an analytical expression to describe the effective contact radius evolution; and third, use this corrected contact radius description in the corresponding analytical indentation model to fit experimental AFM force spectroscopy data. In this way, more robust and physically better-grounded Young’s modulus values can be obtained.

## 4. Conclusions

This study demonstrates that the determination of Young’s modulus of Saccharomyces cerevisiae by AFM is strongly dependent on the description of probe–sample contact. Classical Hertz and Sneddon models were found to significantly underestimate the modulus because they overestimate the contact radius during indentation. Finite element analysis showed that the real contact radius evolves with indentation and remains smaller than that predicted by classical formulations. By incorporating an FEA-derived effective contact radius into these models, a consistent Young’s modulus value of 0.17 MPa was obtained for both spherical (2 µm) and flat (4 µm) probes, whereas unmodified models yielded strongly underestimated and probe-dependent values of 0.027 MPa and 0.006 MPa, respectively.

FEA models of yeast cells with three distinct diameters were developed to investigate the influence of cell size on AFM-based mechanical property estimation. A cell diameter of 6 µm was selected as the experimentally relevant geometry, representing the most common yeast cell size considered in this study. Additional models with diameters of 8 and 10 µm were introduced to evaluate whether natural variations in yeast cell size affect contact-radius evolution and, consequently, the Young’s modulus estimated using classical Hertzian and Sneddon formulations with spherical and flat probes. This analysis allows the error contribution associated solely with cell-size variation to be assessed under otherwise identical indentation conditions. In this context, the key question is whether cell size itself can introduce measurable bias in the final modulus estimation. By comparing the classical contact-model predictions with the contact evolution obtained from FEA, this study also proposes a solution to reduce such errors. The results are expected to provide a broader understanding of how probe-specimen contact mechanics influence the reliability of AFM-based mechanical characterization of soft biological specimens.

Overall, the findings indicate that the apparent variability of yeast mechanical properties is largely methodological rather than purely biological. The proposed modification of classical contact models using FEA-derived contact evolution provides a practical, physically grounded approach to achieving a more accurate, probe-independent determination of Young’s modulus in living cells.

Future research should extend the proposed AFM-FEA correction approach to other probe geometries, indentation ranges, and biological samples with different cell-wall or tissue-like structures. This would allow evaluation of the broader applicability of the fitted contact-radius dependencies and support development of more standardized protocols for AFM-based mechanical characterization.

## Figures and Tables

**Table 1:** Characteristics of cantilevers [18,19,30,31].

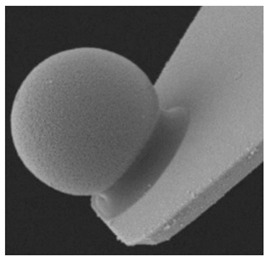	SPHERE: CP-FM-SiO-A-5. sQube (Sofia, Bulgaria). Geometry: Spherical. Dimensions: T = 3 ± 1 µm, L = 225 ± 10 µm, W = 28 ± 7.5 µm. Spring constant: 0.5–9.5 N/m. Measured: 4.18 N/m. f0 = 45–115 kHz. Tip radius: 2 µm ± 5%.
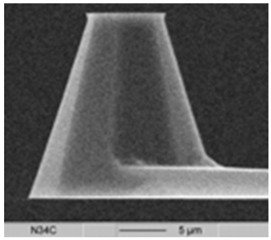	FLAT: SD-PL-FM-10. Nanosensors (Neuchâtel, Switzerland). Geometry: Rectangular. Dimensions: T = 3 ± 1 um, L = 225 ± 10 um, W = 28 ± 7.5 µm, f0 = 75 kHz. Spring constant: 2.8 N/m. Measured: 1.47 N/m. Tip plateau diameter 8–12 μm, tip height 10–15 µm.

## Data Availability

The original contributions presented in this study are included in the article. Further inquiries can be directed to the corresponding authors.
